# Developing sustainable capacity-building in mental health research: implementation outcomes of training of trainers in systematic reviewing

**DOI:** 10.1080/16549716.2020.1715325

**Published:** 2020-02-11

**Authors:** Helen E. Jack, Christopher Merritt, Girmay Medhin, Rosemary Musesengwa, Chitsanzo Mafuta, Lorna J. Gibson, Charlotte Hanlon, Katherine Sorsdahl, Dixon Chibanda, Melanie Abas

**Affiliations:** a Centre for Global Mental Health, Institute of Psychiatry, Psychology, and Neuroscience, King’s College, London, UK; b Department of Medicine, University of Washington, Seattle, WA, USA; c Aklilu Lemma Institute of Pathobiology, Addis Ababa University, Addis Ababa, Ethiopia; d Department of Psychiatry, University of Oxford, Oxford, UK; e Department of Mental Health, College of Medicine, University of Malawi, Blantyre, Malawi; f Tropical Epidemiology Group, Faculty of Epidemiology and Population Health, London School of Hygiene and Tropical Medicine, London, UK; g Department of Psychiatry, WHO Collaborating Centre for Mental Health Research and Capacity-Building, School of Medicine, College of Health Sciences, Addis Ababa University, Addis Ababa, Ethiopia; h Centre for Innovative Drug Development and Therapeutic Trials for Africa (CDT-Africa), College of Health Sciences, Addis Ababa University, Addis Ababa, Ethiopia; i Alan J. Flisher Centre for Public Mental Health, Department of Psychiatry, University of Cape Town, Rondebosch, South Africa; j Department of Psychiatry, University of Zimbabwe College of Health Sciences, Harare, Zimbabwe

**Keywords:** Global mental health, pedagogy, sustainability, research capacity building, health system strengthening, evidence-based practice

## Abstract

Less than 1% of biomedical research papers originate in Africa. Locally relevant mental health research, including synthesis of existing evidence, is essential for developing interventions and strengthening health systems, but institutions may lack the capacity to deliver training on systematic reviewing for publication in international journals. This paper describes the development and implementation of a training-of-trainers (ToT) course on systematic reviewing. The ToT prepared junior faculty (‘trainers’) from universities in Ethiopia, Malawi, and Zimbabwe to lead a five-day systematic reviewing workshop. Using an evaluation framework based on implementation science outcomes, the feasibility of the ToT was assessed by tracking the number of workshops the trainers subsequently conducted and the number of trainers and trainees who participated; acceptability was assessed through post-workshop surveys on trainee perspectives; impact was evaluated through trainee scores on a 15-item multiple choice test on systematic reviewing concepts; and sustainability was assessed based on whether the workshop was integrated into university curricula. Twelve trainers (86% of those trained) facilitated a total of seven workshops in their home countries (total 103 trainees). The first workshop run in each country was evaluated, and there was a significant improvement in mean knowledge scores between pre- and post-tests among trainees (*MD*= 3.07, *t*= 5.90, 95% CI 2.02–4.11). In two of the three countries, there are efforts to integrate the systematic review workshop into university curricula. The cost of the workshop led by the international trainer was $1480 per participant, whereas the trainer-led workshops cost approximately $240 per participant. Overall, ToT is relatively new to research capacity building, although it has been used widely in clinical settings. Our findings suggest ToT is a promising, low-cost way to develop both technical skills of individuals and the pedagogical capacity of universities, and to promote sustainability of research capacity building programs that often have time-limited grant funding.

## Background

Research infrastructure is essential for developing locally relevant evidence, evaluating practice, and strengthening health systems [1,2]. In biomedical research broadly, and in mental health research specifically, low- and middle-income countries (LMICs) produce a small fraction of published papers, particularly relative to their burden of disease [3]. The relative paucity of research outputs stems from insufficient financial resources to support research; time constraints of trained professionals; and lack of adequate training in conducting high-quality research [3,4].

In recent years, there has been growing attention to developing mental health research capacity, both through collaborations between high-income countries (HICs) and LMICs, and the formation of African research networks [3,5–7]. Research capacity building has taken a variety of forms, including the development of masters and PhD programs at African institutions [5], mentorship of African researchers [3], integrating research into clinician education [8], and provision of short courses on key research skills [5,9].

While a ‘training of trainers’ (ToT) approach has been widely used to increase the sustainability of a variety of  more clinical global health programs [10,11], including in mental health [12], there are few examples of ToT within research capacity building [5]. One instance of a ToT for mental health research is part of the NIH-funded South Asian Hub for Advocacy, Research, and Education on Mental Health (SHARE) project, which developed a short course on mental health research in humanitarian settings and used a ToT approach to spread that course throughout South Asia, but did not detail the process or outcomes of the ToT [13]. Conducting a ToT that results in sustainable, ongoing training is not without its challenges, including trainers not feeling sufficiently confident to implement the training, lack of resources for subsequent trainings, and poor fidelity to the original curriculum [10]. However, ToT is a potentially viable model for expanding access to research methods training in low-resource settings and as a mechanism for HIC–LMIC partnerships that result in lasting change in LMICs.

Accordingly, we developed a ToT for health research and trained trainers from three African countries to conduct a systematic review workshop at their home universities as part of a broader mental health research capacity-building project. This ToT focused specifically on systematic reviewing skills because being able to conduct a comprehensive literature review is a competency that is broadly useful for researchers, regardless of what methodology they are using to collect primary data. Systematic reviewing teaches foundational research skills of question development, database searching, organization of literature, and analysis of background literature; it also can make research more efficient by helping focus work on areas where there are clear evidence gaps. Our aims in this paper are to describe the ToT and the process of its development and present preliminary feasibility and acceptability results. While controlled testing of outcomes is needed, these findings may inform the development of similar interventions within other research capacity building programs.

## Methods

### The intervention

**Figure 1.**
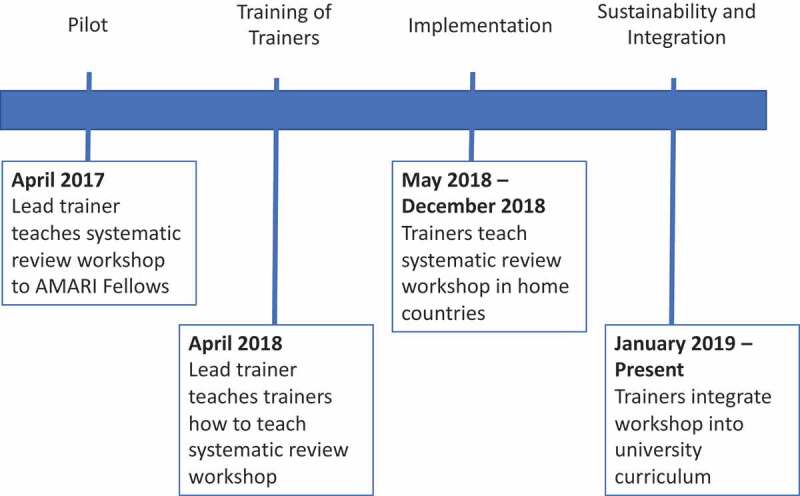
Timeline and four phases of ToT implementation

The African Mental Health Research Initiative (AMARI) supports 19 MPhil students, 24 PhD students, and five post-doctoral researchers conducting mental health research at universities in Zimbabwe, Ethiopia, Malawi, and South Africa (‘AMARI fellows’) [14]. With financial support from AMARI, AMARI provided mentorship from partner institutions in HICs and South Africa and short courses on research methods and leadership. In 2017, a 7-day course on systematic reviewing was taught to the first cohort of AMARI fellows (n = 17) (‘pilot workshop’). The pilot workshop was well-received, with positive student experience reviews and publication of three systematic reviews and three protocols [15–20]. Instead of offering the same workshop to the second cohort of AMARI fellows, the workshop was converted into a ToT that aimed to train senior PhD students and faculty from AMARI institutions to teach the workshop at their home institutions to both AMARI fellows and other trainees. The timeline and phases of the pilot systematic review workshop and subsequent ToT are displayed in Figure 1.

**Caption**: *Terms in this diagram*: AMARI Fellows: PhD and post-docs supported by capacity building grant; Lead trainer: senior researcher who teaches systematic reviewing; Trainers: participants in the Training of Trainers from Zimbabwe, Malawi, and Ethiopia

#### Systematic review workshop curriculum

The pilot systematic review workshop curriculum was delivered by a researcher from King’s College London (HEJ), an AMARI partner institution. The workshop was developed following consultation with instructors at schools of public health in HICs who taught systematic review courses, who provided input on curriculum formats, and in consultation with in-country and external AMARI supervisors, who highlighted key areas for growth among AMARI fellows. A list of modules is displayed in Table 1. Teaching was geared toward providing participants with the tools they needed to complete a systematic review following the Preferred Reporting Items for Systematic Reviews and Meta-Analysis (PRISMA) guidelines [21]. Participants had to be working on a systematic review in order to participate in the workshop. The interactive training centered on participants learning content and building skills through working on their own systematic review. Evidence-based pedagogical techniques, including active learning, project-based learning, and minimizing lecture-style presentations were used throughout the workshop [22] (Table 1).

**Table 1.** Training of trainers

**Systematic review core modules**: *Delivered by trainers in their systematic reviewing workshops and reviewed with and practiced by trainers during the ToT*	
Unit	Description
Setting up your review	What is a systematic review? Question development Registering systematic reviews with PROSPERO (international registry: https://www.crd.york.ac.uk/prospero/)
Conducting and saving searches	Boolean logic Using PubMed Overview of using other databases Working with a reference librarian Accessing full-text articles
Selecting eligible articles	Using a reference manager Developing eligibility criteria Documenting a systematic review search
Methodology assessment	Assessing risk of bias within and across studies
Collecting, analyzing, and writing up data	Data extraction Presentation of results Writing the results section Writing the discussion section
Dissemination of findings	How to share research findings beyond academic publication (policy briefs, advocacy, and popular press)
**Pedagogy strategies**: *Taught to trainers during ToT and modeled during ToT and systematic review workshop*	
Teaching	Description
Active learning	After a brief didactic introduction to a concept, learners do interactive exercises, often in groups, to learn and solidify the concept [28]. For instance, the systematic review workshop used detailed worksheets that stepped participants through creating their own search in groups, rather than a lecture on search development.
Project-based learning	Learners are actively working on their own project and use project work to develop their skills [29]. For instance, all participants in the systematic review workshop had to be working on a systematic review and had devoted time during the workshop to make progress on on their own review.
Minimal lectures	Lectures were kept to a minimum and typically lasted less than 20 minutes, which may help with maintaining attention and emphasizing more active forms of learning [28]. Additionally, some lectures were provided as short concept videos that could be watched online outside of class, leaving time in class for interactive activities [30,31].

#### ToT curriculum

The aim of the ToT was to teach trainers how to deliver the systematic review workshop, described above. For clarity, participants in the ToT will be referred to as ‘trainers’, those they teach will be ‘trainees’, and the teacher of the ToT will be the ‘lead trainer’.

The ToT was conducted over 5 days. Each day consisted of an interactive review of key systematic review content, teaching and exercises about pedagogy, teaching practice, and time to discuss the logistics of delivering the workshop in participants' home countries. Participants taught a two-hour session to local students on the final day of the workshop then received feedback. Teaching practice and logistics discussions were done in groups by country, as participants planned to return to their home countries and deliver the workshop as a team. This protected time for planning was added to give participants an opportunity, free from clinical and research responsibilities, to plan their workshops, and was added in response to prior research showing that lack of time and resources are barriers to trainers in ToTs conducting trainings [10]. During the workshop, trainers were provided with a trainer guide that detailed how to deliver each lesson; PowerPoint presentations for all didactic sessions; handouts that guided trainees through interactive exercises that built toward their systematic review protocol; and short explanatory videos in a digital folder that helped reinforce particularly complex concepts, such as Boolean logic. Trainers were encouraged to modify these materials for their setting.

#### Participants

The ToT was held in Harare, Zimbabwe. The 14 participants were from three countries (Ethiopia, Malawi, and Zimbabwe) and were a combination of PhD students (n = 6), post-docs (n = 1), faculty members (n = 5), reference librarians (n = 1), and program managers (n = 1). Five had PhDs in relevant fields, and the six PhD students were AMARI fellows. All participants were nominated by their institutions and were required to have taken a prior systematic reviewing course and had been involved in a systematic review previously, ideally as the first author. Additionally, preference was given to people who were well-positioned to use the skills gained in the ToT in their ongoing work as academics (both teaching and their own research) and who had roles within their universities that might allow them to implement the systematic review workshop more broadly. All of the participants who were not PhD students were involved in teaching research methods and/or supervising researchers at their home institutions. The PhD students who were selected were far along in their PhDs, many supervised Master’s students, most anticipated pursuing academic jobs where they could continue teaching and research, and some already held faculty appointments at universities.

#### Implementation

On the final day of the ToT, trainers in groups by country gave a presentation on their plan for their workshop, including provisional dates, anticipated challenges to delivery, and proposed content modification for their context. They then returned to their home countries they received financial support from AMARI to deliver their first workshops. The lead trainer remained available to answer questions remotely during the initial workshop delivery. The participants were encouraged to identify mentors at their home institutions (reference librarians, senior faculty) who could answer technical questions and provide professional support during workshop implementation.

### Evaluation of the intervention

We focused on the evaluation of selected implementation science outcomes for the ToT and also present preliminary data on trainee knowledge. Implementation science assesses how an intervention works in a complex, real-world environment, including whether it is feasible, acceptable, affordable and sustainable [23,24].

#### Implementation science

##### Feasibility

Feasibility assesses whether an intervention can be implemented in a new setting, regardless of outcomes [25]. A common challenge of ToTs is that the trainers lack the resources, confidence, or expertise to conduct subsequent trainings [10]. We evaluated whether ToT was feasible by counting the number of workshops conducted, and numbers of trainers teaching and trainees trained within the 8 months following the ToT.

##### Acceptability

Trainers administered a standardized post-workshop survey to all trainees to assess their satisfaction with the workshop and their perception of their confidence conducting a systematic review on a five-item Likert scale.

##### Sustainability

The primary aim of the ToT was to prepare trainers to teach a single 5-day workshop as part of the AMARI programme. However, trainers were encouraged to look for opportunities to implement all or parts of the workshop elsewhere in their universities and, ideally, to integrate it into the permanent curriculum. They were not, however, given specific support to do this. To evaluate sustainability, we solicited input from the groups of trainers at each institution on whether the systematic reviewing workshop would be permanently integrated into the university’s curriculum and, if so, in what form.

##### Cost

We report the cost of each workshop (total and per participant). Costs included venue, food, training supplies, and in some cases, transportation for participants from their home countries, stipends for participants or trainers, and accommodation for participants who were not from the city where the workshop was held.

#### Impact

##### Trainee knowledge

All trainees took a 15-question multiple-choice test before and after the workshop. The test included questions about aspects of systematic reviewing, including developing a question, building a search strategy, screening studies for inclusion, assessing the risk of bias, and writing a manuscript. An unpaired t-test was used to determine whether there was a difference between pre and post-intervention mean scores.

## Results

#### Implementation science

##### Feasibility

The trainers facilitated at least one workshop in each country. Where more than one workshop was taught, only the first workshop taught in each country was included in this analysis. Eighty-six percent (12/14) of the trainers trained helped lead workshops. They trained a total of 30 trainees (17 from Ethiopia, 5 from Malawi, and 8 from Zimbabwe), who were primarily PhD and MPhil students, post-doctoral researchers, and junior faculty members from the universities where the trainings were conducted.

##### Acceptability

While 30 trainees participated in the workshops, only 26 completed the post-workshop survey (Table 3). All four participants who did not complete the post-workshop survey were from Zimbabwe. One participant from Ethiopia did not complete the confidence part of the post-workshop survey. Over 90% of trainees either agreed or strongly agreed with all assessments of satisfaction except that the length of the course was sufficient (6 trainees, 23%, felt either neutral or disagreed that the length was sufficient). Notably, 25/26 trainees believed that the trainer had sufficient expertise in the training content. Over 88% of trainees felt confident that they had the skills and mentorship to complete a systematic review and had a plan to complete their review (Table 2).

**Table 2.** Post-intervention confidence and satisfaction survey results

Question	Strongly Disagree	Disagree	Neutral	Agree	Strongly Agree	Total of respondents
The content of the course was consistent with what I had expected.	0	0	2 (7.7%)	5 (19.2%)	19 (73.1%)	26
The facilitators had sufficient expertise on the training content.	0	0	1 (3.8%)	17 (65.4%)	8 (30.8%)	26
The training was relevant to my knowledge needs.	0	0	1 (3.8%)	1 (3.8%)	24 (92.3%)	26
The sessions were sufficiently interactive.	0	0	0	2 (7.7%)	24 (92.3%)	26
The length of the course was sufficient.	0	3 (11.5%)	3 (11.5%)	9 (34.6%)	11 (42.3%)	26
The facilitators were always responsive to my needs.	0	0	0	7 (26.9%)	19 (73.1%)	26
I feel confident that I have the skills to complete a systematic review.	0	0	1 (4.0%)	19 (76.0%)	5 (20.0%)	25
I feel confident that I have created a plan to complete the systematic review that I started in this workshop.	0	0	3 (12.0%)	15 (60.0%)	7 (28.0%)	25
I have identified sources of mentorship and support for completing my systematic review.	0	0	1 (4.0%)	17 (68.0%)	7 (28.0%)	25

**Table 3.** Cost of ToT and subsequent workshops

Workshop	Cost*	Number of participants	Per participant cost
Systematic review workshop (pilot, 7 days)	$25,169	17	$1480.53
Training of Trainers (5 days)	$12,101.73	14	$864.41
Malawi	$1200	5	$240
Zimbabwe	$1920	8	$240
Ethiopia	$4000	17	$235.29

* All costs include venue, food, transportation (if necessary), accommodation (if necessary), training supplies, and stipends for participants and trainers when relevant.

##### Sustainability

In Ethiopia, the trainers offered to deliver the workshop within and outside of their university to interested groups (departments, other universities). Those outside departments paid all training costs. The trainers from Ethiopia conducted four workshops (77 participants) in the year following the ToT. Additionally, the trainers from Ethiopia worked with their university to integrate the systematic review workshop into their Masters’ in Clinical Trials. In Zimbabwe, trainers are working with their university to have aspects of the workshop incorporated into post-graduate training in Research Methodology. The workshop has not been replicated in Malawi, and there are no plans for subsequent workshops.

##### Cost

Data on the cost of each workshop and number of participants is reported in Table 3. The total cost of the ToT was $12,101.73 ($864 per participant). Costs of the trainer-led workshops ranged from $235-$240 per participant, whereas costs for the initial workshop led by an international trainer was $25,169 ($1480.53 per participant).

### Impact

#### Trainee knowledge

Two participants did not complete the post-test and one did not complete both pre and post-tests, leaving a sample of 26 participants with complete data for analysis. The mean difference (post – pre) was 3.07 (*t*= 5.90, 95% CI 2.02–4.11) (Table 4).

**Table 4.** Unpaired samples test

	Number of observations	Mean	Std. deviation	Std. error of the mean	95% CI	t	Degrees of freedom
Pre-test	26	10.08	2.33	0.46	9.13–11.02		
Post-test	28	13.14	1.41	0.27	12.60–13.69		
Difference		3.07		0.52	2.02–4.11	5.90	52

## Discussion

This paper describes the implementation and outcomes of a ToT for teaching systematic reviewing to early career researchers in sub-Saharan Africa. Overall, the ToT was feasible and acceptable and is being integrated into curricula at two out of the three universities. Additionally, the ToT was substantially lower cost per participant trained than the workshop led by an international trainer ($240 for ToT versus $1480 per participant for the international trainer). Preliminary, non-controlled data shows that the ToT resulted in improved trainee knowledge in systematic reviewing, but further, more rigorous evaluation is needed. This is among the first papers to describe a ToT intervention for research methods in LMICs, a possible mechanism for enhancing the sustainability of grant-funded capacity-building efforts.

This study responds to calls in existing research for greater emphasis on ToT programs within mental health capacity building efforts [5,12] and adds to the growing evidence base on research capacity building in LMICs. We found an increase in knowledge and subjective reports of high confidence, findings echoed in other studies on capacity building [5,13]. Like many research capacity-building programs, our evaluation was limited to proximal outcomes, such as confidence and knowledge, rather than more meaningful, distal outcomes, such as publications, grants, career trajectories of participants, and change in policy or practice [26]; these should be a focus of future research. Additionally, we used a pre-post assessment of knowledge, without a control group, which means that we cannot assess causality. However, given the lack of research on ToTs for research methods, we believe that sharing our process of workshop development and implementation may provide ideas and a blueprint for future programs and randomized, controlled studies.

Mormina et al. have proposed a framework for the evaluation of ToT interventions. The framework emphasizes the importance of alignment between the objectives of the ToT intervention and the trainers’ professional goals [11]. Because the trainers in our intervention were aspiring or current academics, teaching research skills was part of their jobs and career goals, perhaps helping explain why trainings were successfully implemented in all three countries and were sustained in two of three. Other principles of the framework include talent (of the lead trainer and trainers), resources (to conduct the trainings), implementation (whether future trainings were implemented), and nurturing (ongoing training and support). Talent and resources were strengths of our program, as most trainers had experience writing systematic reviews, and there was an external capacity-building grant that supported the initial ToT and subsidized subsequent workshops. Resources to conduct future iterations of the workshop will become more difficult when AMARI funding ends. This study, however, showed that the in-country workshops were far less costly per participant than the initial pilot workshop taught by an international trainer, and some trainers were able to find local support for ongoing trainings. Nurturing was a challenge, as ongoing coaching was provided remotely by the lead trainer and only if trainers had specific questions, which may have limited the fidelity to the original curriculum.

We can draw a number of lessons for future capacity-building efforts from our experience. First, the ToT included trainers of a variety of levels within the academic hierarchy from each institution (from PhD students to faculty members). The diversity in levels of trainers facilitated trainers having different strengths and taking on specific roles in training and workshop organization. Junior faculty were tasked with logistical arrangements and course preparations such as advertising, selection of candidates, and preparation and distribution of course material. Senior faculty provided research and teaching guidance and were tasked with helping integrate of the course into the postgraduate training programs. Second, both the ToT and the workshop curriculum used evidence-based pedagogical practices, such as interactive, project-based learning and few lectures. Discussion of pedagogical techniques is relatively new to the ToT literature [10] and more research is needed to explore how pedagogy affects ToT outcomes. Future studies could use observed teaching sessions to evaluate fidelity to these evidence-based pedagogical practices, as fidelity is an important component of effective implementation and scale-up. Third, overall, the ToT was sustainable, being integrated into curricula at two out of the three universities. One key reason for the sustainability may have been that the ToT provided comprehensive teaching materials (Powerpoints, handouts, trainer guide), which made it easier for trainers to deliver the workshop without substantial additional preparation. This may be particularly important in low-resource settings, where many trainers have clinical and teaching responsibilities that they have to balance with development of any new curriculum. The workshop was implemented widely in Ethiopia, which may have been due to both local demand (many departments interested in funding trainings) and trainer interest and enthusiasm. Because the funding for the ToT was part of a grant that aimed to build capacity for a specific group of individuals (AMARI fellows), the ToT primarily sought to prepare trainers to teach a single five-day workshop to AMARI fellows. Its primary aim was not to equip trainers to integrate the workshop into existing curricula, which would have necessitated an approach that more fully involved key people involved in curriculum planning at each institution and ongoing support for curriculum development. However, the fact that many ToT participants chose to integrate the workshop into permanent curricula without being given support or being incentivized to do so further underscores the local acceptability of the workshop. Fourth, both during the ToT and during the subsequent workshops, there were challenges with internet connectivity and academic database access, a stark inequity between LMIC and HIC institutions. A role for HIC partner institutions may be providing database access and helping support institutional internet connectivity, as has happened in the AMARI programme. Although there are a growing number of open-access journals and many LMIC institutions gain database access through Hinari, a WHO-run program [27], more still needs to be done to ensure that LMIC researchers can access scientific knowledge on an equal footing.

This evaluation has a number of limitations. First, while we evaluated trainee knowledge, we did not evaluate more meaningful, distal outcomes, such as systematic reviews published in peer-reviewed journals or practice change from systematic review findings, because of the short follow-up time and the anticipated long delay between training, publication, and subsequent practice change. We chose to evaluate knowledge because it is necessary for completing a systematic review, but recognize that it is clearly not sufficient. Accordingly, longer-term evaluation of this and similar trainings is important to develop an evidence-base for global health capacity-building efforts. We intend to track the systematic reviews published by participants in these workshops if our consortia’s bid, currently in progress, is funded to extend AMARI for a further five years. Second, we did not evaluate knowledge using a validated tool, but designed a test that specifically evaluated the content taught in the ToT. It was reassuring that participants had relatively similar test scores to each other and that the test was sufficiently challenging that participants did not have uniformly high scores at pre-training that would have made it challenging to look for improvement. Notably, however, the focus of this evaluation was not to evaluate outcomes but to describe the development and implementation of the workshop to be an example for future researchers and practitioners. Third, we did not assess fidelity to the original training. While poor fidelity to the original intervention is a frequently highlighted challenge of ToTs [10], most ToTs prepare lower-skilled healthcare workers to deliver trainings. This ToT was distinct, as it was training PhD-level research professionals, who have the background and qualifications to modify the materials for their settings. Consequently, it may not have been appropriate to measure fidelity to the details of the curriculum, but it would be helpful to assess fidelity to evidence-based pedagogical practices and core components of the curriculum. Such fidelity investigation could also reveal changes that trainers made to fit the needs of their trainees and the context, helping with adaptation of future trainings and assessing whether further strengthening of the ToT was needed if trainers across settings modified a common component.

## Conclusions

Investment in research capacity building is one way to begin to build-up systems to help close the health research gap in LMICs . This study shows that ToT interventions may be an approach to expand access to research capacity building in a way that empowers LMIC partners, giving them ownership over capacity-building, and may be more sustainable than interventions taught only by HIC trainers. Future research should track longer-term outcomes of ToT interventions and examine how different pedagogical strategies affect these outcomes.

## Paper context

Only a small fraction of biomedical research comes from African institutions, despite the continent’s large burden of disease. As part of a research capacity-building project, an international trainer taught a group of local trainers to deliver a systematic reviewing workshop, which they then implemented in three African countries. Here, we describe the evaluation of training of trainers (ToT) using implementation science principles. Overall, the ToT was feasible, acceptable, low cost, sustainable, and resulted in increased knowledge.
